# The Response of Creatine Kinase Specific Activity in Rat Pituitary to Estrogenic Compounds and Vitamin D Less-Calcemic Analogs

**DOI:** 10.1155/2009/273651

**Published:** 2009-09-10

**Authors:** D. Somjen, N. Mirsky, S. Tamir, J. Vaya, G. H. Posner, A. M. Kaye

**Affiliations:** ^1^Institute of Endocrinology, Metabolism and Hypertension, Sourasky Medical Center, Tel-Aviv 64239, Israel; ^2^The Sackler Faculty of Medicine, Tel-Aviv University, Tel-Aviv 64239, Israel; ^3^Faculty of Science, University of Haifa, Har-Hacarmel, Haifa 31905, Israel; ^4^Laboratory of Natural Compounds for Medical Use, Migal-Galilee Technological Center, Kiryat- Shmona 10200, Israel; ^5^Department of Chemistry, The Johns Hopkins University, Baltimore, MD 21218, USA

## Abstract

We examined the response of rat female pituitary at different metabolic stages to treatments with estrogenic compounds and vitamin D analogs. Immature or ovariectomized (Ovx) female rats responded by increased creatine kinase specific activity (CK) to estradiol-17*β* (E_2_), genistein (G), daidzein (D), biochainin A (BA), quecertin (Qu), carboxy- G (cG), carboxy- BA (cBA), and raloxifene (Ral). The response was inhibited when Ral was injected together with the estrogens. CK was increased when hormones were injected daily into Ovx rats for 4 different time periods. Pretreatment with the less-calcemic vitamin D analogs JK 1624 F_2_-2 (JKF) or QW 1624 F_2_-2 (QW) followed by estrogenic injection resulted in increased response and sensitivity to E_2_ and loss of inhibition of E_2_ by Ral. CK was also increased by feeding with E_2_ or licorice or its components dose- and time- dependent in immature or Ovxrats. Diabetic female rats did not respond to increased doses of E_2_. In conclusion, rat female pituitary is estrogens-responsive organ, suggesting to considerits response for HRT in postmenopausal women for both beneficial and hazardous aspects.

## 1. Introduction

Estradiol-17*β* (E_2_) is essential for all aspects of reproductive function in females through activation of estrogen receptors (ERs). In the rat E_2_ stimulates basal secretion of pituitary reproductive hormones [1]. The pituitary expresses both estrogen receptors, ER*α* and ER*β* [2], and it responds to both ER*α* specific and ER*β* specific agonists [33]. E_2_ was found to have pleiotropic effects on physiological function in rat pituitary [3].

 Phytoestrogens are plant-based estrogenic compounds, which are selective estrogen receptor modulators (SERMs) due to their ability to induce both agonistic and antagonistic effects. There is a growing interest in the use of phytoestrogens in Western countries. Widely marketed as food additives and present at fairly high concentrations in soy products [4, 5], phytoestrogens are commonly treated in the lay media as a uniform class of naturally occurring estrogenic compounds retaining the beneficial effects of estrogens but carrying none of the harms potentially inflicted by native or synthetic estrogens. However, phytoestrogens vary considerably in terms of structure, estrogenic potency, and availability in common food sources such as soybeans, cereals, and sprouts [5]. Most human dietary sources contain phytoestrogens of two major chemical classes, isoflavones and lignans. The isoflavone genistein (G) is perhaps the best studied phytoestrogen [5–7] whereas data on the biological effects of other common isoflavones such as daidzein (D) or its metabolite equol [5–7] are relatively scarce. Based on favorable effects of these compounds on lipid oxidation [8, 9] and vascular reactivity [5], a recent review of literature suggested that dietary phytoestrogen consumption may confer cardiovascular protection [6]. Phytoestrogens in the diet were also found to affect pituitary of rats, by modulating serum gonadotropin levels [10] similar to E_2_.

 Licorice root extract (L) and its major isoflavans, glabridin (Gla) and glabrene (Glb), exhibited varying degrees of ERs' agonism in different tissues in vivo. Animals fed with L, Gla, and Glb similar to E_2_ showed increased CK in different organs [11, 12]. Phytoestrogens fed to rats were also found to alter some neurobehavioral effects [13] similar to E_2_.

 Vitamin D binding protein is expressed in rat hypothalamus which shows its biological activity [14]. Vitamin D receptors (VDR) are also expressed in the hypothalamus [15] as well as in human pituitary [16] and in rat pituitary [17–19].

E_2_ regulates a spectrum of activities in the pituitary as well as induction of calbindin D9K in the rat [20] which is also regulated by vitamin D metabolite 1,25(OH)_2_D_3_ [21].

 We have previously studied the hormonal modulations in different systems of the specific activity of the “estrogen-induced protein” creatine kinase BB [22], a rapid estrogen response-marker.

 The present study was undertaken to see if, due to the presence of both E_2_ and vitamin D receptors, the pituitary of female rats responds by induction of CK, to different hormones such as E_2_ and different phytoestrogens with and without pretreatment with the less-calcemic vitamin D analogs JKF and QW by different ways of application and at different physiological status. The obtained results might suggest considering also the response of the pituitary to hormonal treatment, such as hormone replacement therapy (HRT) for postmenopausal women, is used for both its beneficial and its hazardous aspects.

## 2. Materials and Methods

**Rats**

(1) Wistar-derived prepubertal female rats, aged 25 days weighing 60 g at the start of the experiment (intact) or 2 weeks postovariectomy (Ovx), were used. Intact or Ovx rats were injected daily (5 days per week) for 10 weeks with either E_2_ (5 *μ*g/rat), Ral, G, cG, cBA, BA, or D (all at 500 *μ*g/rat), or raloxifene (Ral 500 *μ*g/rat) or all hormones with Ral, or all pytoestrogens together with E_2_. In other experiments, licorice (L, 25 *μ*g/rat), or its synthetic derivatives glabridine (Gla, 25 *μ*g/rat) or glabrene (Glb 25 *μ*g/rat) with and without E_2_ or Ral was given to rats by feeding for different time periods. The doses used were found in previous studies, to be the optimal effective doses in this model.

(2) Sprague Dawley female rats, at the age of 5 weeks, weighing 120 g, was injected subcutaneously with a single dose of Streptozotocin (STZ; 60 mg/Kg BW in 0.05 M citrate buffer, pH 5.7). Additional group of animals were injected with the vehicle (0.05 M citrate buffer, pH 5.7) and served as healthy controls. The animals were kept for 8 weeks in cages with 12 hours cycles of light and dark, Purina chow and tap water supplemented ad libidum [23]. Rats were used either as intact or 4 weeks postovariectomy (Ovx). E_2_ at different doses was injected for 24 hours, followed by harvesting the pituitary for creatine kinase specific activity (CK) assay.

(3) For pretreatment with vitamin D less-calcemic analogs, Wistar-derived rats were used at initial age of 25 days. Female rats were used either as intact or after ovariectomy (Ovx), and treatments started 2 weeks postsurgery. Rats were injected daily for different time periods as indicated with the analogs JKF 1624F_2_-2 (JKF) or QW 1624F_2_-2 (QW) (0.2 ng/gr BW), and 24 hours after the last injection, rats were injected with E_2_ (0.5 *μ*g/rat), raloxifene (Ral) (500 *μ*g/rat) or both followed by harvesting the pituitary for creatine kinase specific activity (CK), assay.

**Creatine Kinase Specific Activity Preparation and Assay**

Rat pituitary was collected and homogenized in cold isotonic extraction buffer using a Polytron homogenizer. Enzyme extracts were obtained by centrifugation of homogenates at 14000 × g for 5 minutes at 4°C. CK specific activity was measured in a Kontron Model 922 Uvicon Spectrophotometer using a Sigma coupled assay kit, and protein was assayed by Coomassie brilliant blue dye binding. Results are means ± SEM and are expressed as % of control of CK in hormone-treated compared to vehicle-treated, control animals.

**Materials**

Estradiol-17*β* (E_2_), creatine kinase (CK) assay kit, and all phytoestrogens used were purchased from Sigma Chemicals Co. (St. Louis, MO. USA). Raloxifene (Ral) was donated by Dr. B. Founier, Novartis Basel Switzerland. The carboxy-derivatives of the phytoestrogens were synthesized by us [24, 25]. Licorice, glabridin, and glbrene were prepared by us from the roots of G. glabra [26, 27]. JK 1624 F_2_-2 (JKF) and QW 1624F_2_-2 (QW) were synthesized and provided by Dr. G. H. Posner, Johns Hopkins University Baltimore MD. USA [28]. All other reagents used were of analytical grade.

**Statistical Analysis**

Data were calculated as % stimulation by the treatment relative to control rats for each experiment as previously described. Comparison between the control and various treatments was made by analysis of variance using ANOVA.

## 3. Results

**Effects of Different Estrogenic Compounds on CK Specific Activity in Immature and in Ovx Female Rats**

**Figure 1:**
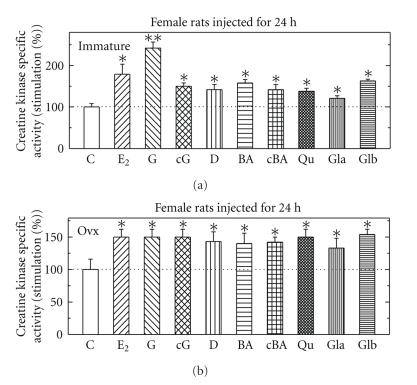
Stimulation of creatine kinase (CK) specific activity by different estrogenic compounds in pituitary in immature female rats (a) and ovariectomized female rats (b). Rats were treated and assayed for CK activity as described in Section 2. Results are means ± SEM for *n* = 5–15 rats/group. Experimental means compared to control means: *P < .05 and **P < .01. Basal activity in pituitary from intact rats was 1.12 + 0.17 *μ*mol/min/mg protein and in Ovx rats was 0.47 + 0.02 *μ*mol/min/mg protein.

When intact female (a) or Ovx (b) female rats were injected for 24 hours with either E_2_ (5 *μ*g/rat), genistein (G), carboxy-G (cG), biochainin A (BA), carboxy- BA (cBA) or daidzein (D) (all at 500 *μ*g/rat), glabridin (Gla) (25 *μ*g/rat), or glabrene (Glb) (25 *μ*g/rat). All hormones that induced CK for different extent in pituitary from both type of animals (Figure 1). Of note is the fact that most of the hormones were more active in pituitary from immature rats than in organs from Ovx rats.

**Effects of Different Estrogenic Compounds with Raloxifene on CK Specific Activity in Immature and in Ovx Female Rats**

**Figure 2:**
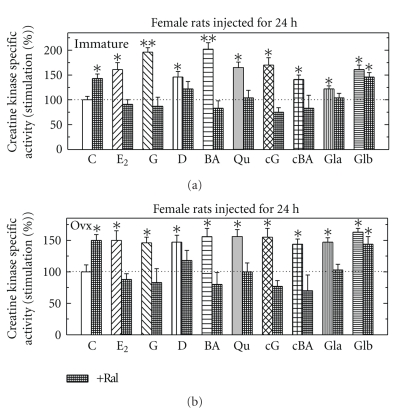
Stimulation of creatine kinase (CK) specific activity by different estrogenic compounds with and without raloxifene (Ral; cross-hatches bars) in pituitary from immature (a) and from ovariectomized female rats (Ovx; b). Rats were treated and assayed for CK activity as described in Section 2. Results are means ± SEM for *n* = 5–15 rats/group. Experimental means compared to control means: *P < .05 and **P < .01. Basal activity in Pi from intact rats was 1.22 + 0.27 *μ*mol/min/mg protein and from Ovx was 0.67 + 0.22 *μ*mol/min/mg protein.

Intact or Ovx female rats were injected for 24 hours with either E_2_ (5 *μ*g/rat), genistein (G), carboxy-G (cG), biochainin A (BA), carboxy- BA (cBA) or daidzein (D) (all at 500 *μ*g/rat), glabridin (Gla) (25 *μ*g/rat) or glabrene (Glb) (25 *μ*g/rat) or raloxifene (Ral 500 *μ*g/rat) or all hormones with Ral. All hormones induced CK for different extent and enzyme induction in the pituitary by all compounds except Glb were inhibited when Ral was injected together with them (Figure 2).

**Effects of Different Estrogenic Compounds with and without Estradiol-17*β* on CK Specific Activity in Immature and in Ovx Female Rats**

**Figure 3:**
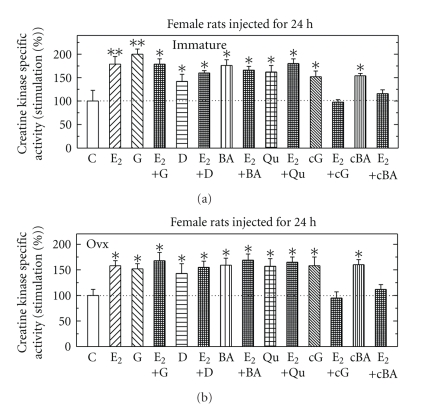
Stimulation of creatine kinase (CK) specific activity by different estrogenic compounds with and without E_2_ in pituitary from immature (a) and from ovariectomized female rats (Ovx; b). Rats were treated and assayed for CK activity as described in Section 2. Results are means ± SEM for *n* = 5–15 rats/group. Experimental means compared to control means: *P < .05 and **P < .01. Basal activity in organs from immature and OVX rats were 1.02 + 0.15 *μ*mol/min/mg protein and 0.67 + 0.22 *μ*mol/min/mg protein, respectively.

Intact or Ovx female rats were injected for 24 hours with either E_2_ (5 *μ*g/rat), genistein (G), carboxy-G (cG), biochainin A (BA), carboxy- BA (cBA) or daidzein (D) (all at 500 *μ*g/rat), or all hormones with E_2_. All hormones tested induced CK for different extent, but only cG and cBA when injected together with E_2_ inhibited CK induced by each of them alone or E_2_ alone (Figure 3).

**Effects of Different Estrogenic Compunds Injected Daily for 4 Months on CK Specific Activity in Ovx Female Rats**

**Figure 4:**
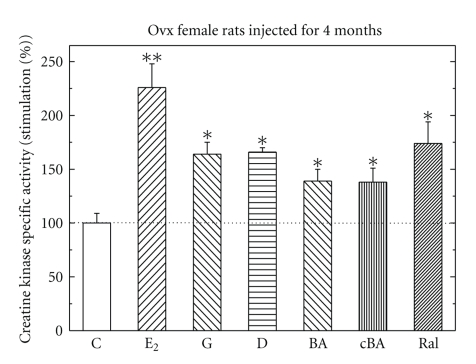
Stimulation of creatine kinase (CK) specific activity by different estrogenic compounds in pituitary from ovariectomized female rats, injected daily for 4 months. Rats were treated and assayed for CK activity as described in Section 2. Results are means ± SEM for *n* = 5–10 rats/group. Experimental means compared to control means: *P < .05 and **P < .01. Basal activity in the pituitary was 0.87 + 0.17 *μ*mol/min/mg protein.

Ovx female rats were injected daily for 4 months with either E_2_ (5 *μ*g/rat), genistein (G), biochainin A (BA), carboxy- BA (cBA) or daidzein (D) (all at 500 *μ*g/rat), or raloxifene (Ral 500 *μ*g/rat). All hormones tested induced CK for different extents (Figure 4). Maximal stimulation was obtained by E_2_ whereas all other phytoestrogens had lower but similar effects.

**Effects of Licorice Fed into Immature Female Rats at Different Doses on CK Specific Activity**

**Figure 5:**
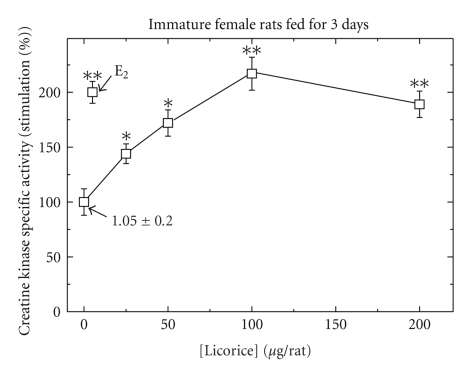
Dose-dependent stimulation of creatine kinase (CK) specific activity by licorice, fed daily for 3 days, in pituitary from immature female rats, compared to feeding with single dose of E_2_. Rats were treated and assayed for CK activity as described in Section 2. Results are means ± SEM for *n* = 5 rats/group. Experimental means compared to control means: *P < .05 and **P < .01. Basal activity was 1.05 + 0.20 *μ*mol/min/mg protein.

When intact immature female rats were fed daily for 3 days with either E_2_ (5*μ*g/rat) or licorice (L) at different doses (25–200 *μ*g/rat), the compounds induced CK dose-dependently (Figure 5). The daily feeding for 3 days with L was maximal at around 100 *μ*g/rat, and this was similar to the stimulation by E_2_ at 5 *μ*g/rat.

**Effects of Licorice or Glabridin or E_2_ Injected into Immature or Ovx Female Rats on CK Specific Activity**

**Figure 6:**
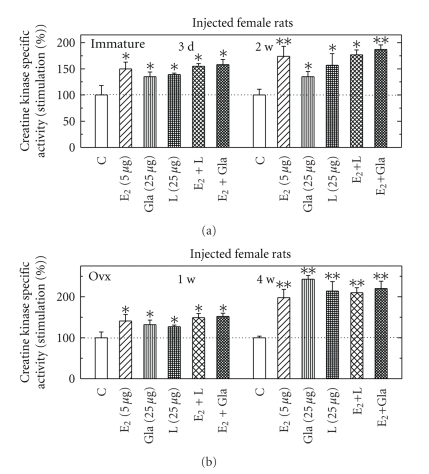
Stimulation of creatine kinase (CK) specific activity by licorice (L), glabridin (Gla) with and without E_2_ in pituitary from immature (a) or from Ovx female rats (b). Rats were fed for different time periods as indicated and assayed for CK activity as described in Section 2. Results are means ± SEM for *n* = 5–10 rats/group. Experimental means compared to control means: *P < .05 and **P < .01. Basal activity in Pi from immature rats was 0.97 + 0.22 *μ*mol/min/mg protein and from Ovx rats was 0.67 + 0.18 *μ*mol/min/mg protein.

When intact or Ovx female rats were injected with either E_2_ (5 *μ*g/rat), glabridin (Gla 25 *μ*g/rat) or licorice (L 25 *μ*g/rat), or E_2_ together with Gla or L, for different time periods, there was an increase of CK by all hormonal combinations injected, with no additivity with the combined treatments at both animal types (Figure 6). In both animal types most of the effects were time-dependent with increased response at the longer time period measured. When Ovx female rats were injected daily for 4 weeks with either E_2_ (5 *μ*g/rat), glabrene (Glb 25 *μ*g/rat) or E_2_ together with Glb, all hormones induced CK for different extent, and when injected together with E_2_, CK induction by E_2_ was inhibited by Glb (data not shown).

**Effects of Different Doses of Estradiol-17*β* for 24 hours on CK Specific Activity in Immature Female Rats Either Intact Or Diabetic**

**Figure 7:**
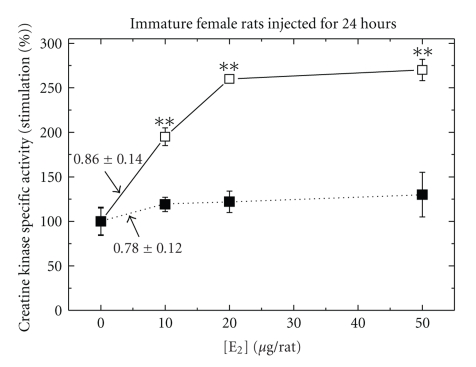
Dose-dependent stimulation of creatine kinase (CK) specific activity by E_2_ in pituitary from immature female rats either intact (solid line) or STZ-injected animals (dotted line). Rats were treated and assayed for CK activity as described in Section 2. Results are means ± SEM for *n* = 5 rats/group. Experimental means compared to control means: *P < .05 and **P < .01. Basal activity in Pi from immature intact rat was 0.86 + 0.04, and from STZ rats was 0.78 + 0.12*μ*mol/min/mg protein.

In intact or Ovx female rats either at their normal or diabetic stage injected for 24 hours with E_2_ (5 *μ*g/rat), CK was induced for different extents only in intact rats but not in diabetic rats [23]. The response to E_2_ which was not induced in the diabetic status was not seen even when higher doses up to 50 *μ*g/rat of E_2_ were used (Figure 7).

**Effects of Estradiol-17*β* with and without Vitamin D Less- Calcemic Analogs on CK Specific Activity in Immature Female Rats**

**Figure 8:**
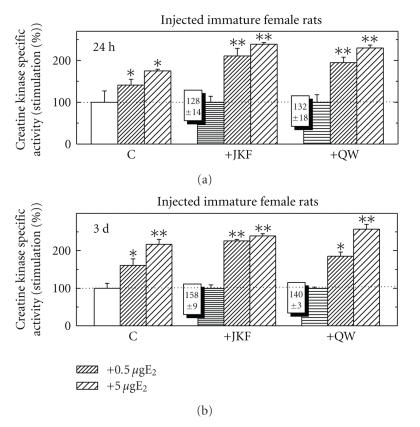
The effect of pretreatment with JKF and QW at 0.2 ng/rat for 24 hours (a) or for 3 days (b) on CK activity in Pi from immature female rats compared to the stimulation of E_2_ at 0.5 *μ*g/rat or 5 *μ*g/rat and to the combination of the vitamin D analogs with E_2_. Treatments were as described in Section 2. Results are expressed as the ratios between the specific activities of CK in hormone-treated and vehicle-injected control animals. The basal activity of CK in was 0.86 + 0.14 *μ*mol/min/mg protein after 24 hours and 0.92 + 0.18 *μ*mol/min/mg protein after 3 days, *n* = 5. *P < .05, **P < .01 in the difference compared to vehicle.

When intact female rats were injected for 24 hours with either E_2_ (at 0.5 *μ*g/rat or 5 *μ*g/rat) alone or with the less- calcemic vitamin D analogs JKF or QW (0.2 ng/gr BW) or daily for 3 days with the analogs followed by E_2_ for 24 hours, all hormones induced CK when injected alone; after pretreatment with JKF or QW the response to E_2_ was up-regulated by about 50% (Figure 8). When rats were injected with E_2_ at 0.5 *μ*g/rat, CK was up-regulated to even higher extent, indicating not only up-regulation of the response to E_2_ but also increased sensitivity (Figure 8).

**Effects of Estradio-17*β* with and without Vitamin D Less-Calcemic Analogs for Different Time Periods on CK Specific Activity in Ovx Female Rats**

**Figure 9:**
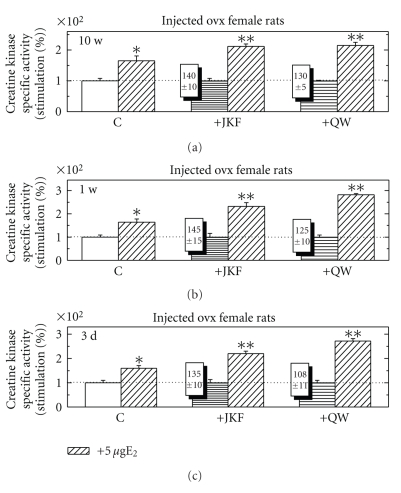
The effect of pretreatment with JKF and QW at 0.2 ng/g/rat/d for 10 weeks (a), 1 week (b) or 3 days (c) on CK activity in Pi from OVX female rats compared to the stimulation of E_2_ at 5 *μ*g/rat and to the combination of the vitamin D analogs and E_2_. Treatments were as described in Section 2. Results are expressed as the ratios between the specific activities of CK in hormone-treated and vehicle-injected control animals. The basal activity of CK in was 0.76 + 0.08 *μ*mol/min/mg protein, *n* = 5. *P < .05, **P < .01 in the difference compared to vehicle.

When Ovx female rats were injected daily for 3 days, 1 week or 10 weeks with the less- calcemic vitamin D analogs JKF or QW (0.2 ng/gr BW) and then E_2_ (5 *μ*g/rat) for 24 hours, CK was induced for different extent by the different hormones. The response of the E_2_was up-regulated by about 2 folds (Figure 9). The highest up-regulation was noticed already after pretreatment for 3 days.

**Effects of Estradiol-17*β* Together with Raloxifene with and without Vitamin D Less- Calcemic Analogs on CK Specific Activity in Immature or Ovx Female Rats**

**Figure 10:**
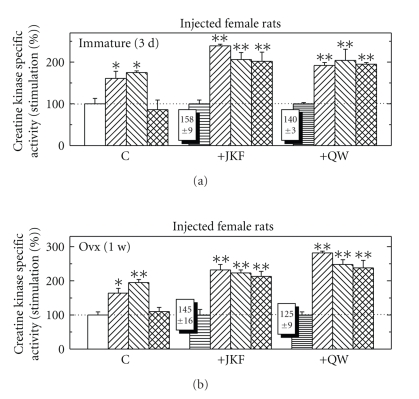
The effect of pretreatment with JKF and QW at 0.2 ng/g/rat/day for 3 days in immature female rats (a) or 1 week in Ovx female rats (lower panel) on CK activity compared to the stimulation by E_2_ at 5 *μ*g/rat or Ral at 500 *μ*g/rat or both, and to the combination of the vitamin D analogs with E_2_, Ral, or both. Treatments were as described in Section 2. Results are expressed as the ratios between the specific activities of CK in hormone-treated and vehicle-injected control animals. The basal activity of CK in was 0.78 + 0.10 *μ*mol/min/mg protein for Ovx and 1.02 + 0.12 *μ*mol/min/mg protein for immature female rats, *n* = 5. *P < .05, **P < .01, in the difference compared to vehicle.

Intact female rats were injected for 24 hours with either E_2_ (5 *μ*g/rat) or Ral (500 *μ*g/rat), or E_2_ and Ral alone, or after daily injections for 3 days with the less-calcemic vitamin D analogs JKF or QW (0.2 ng/gr BW). All hormones induced CK when injected alone, but when E_2_ was injected together with Ral, CK induced by E_2_ was inhibited. After pretreatment with JKF or QW by daily injections for 3 days followed by E_2_, Ral, or E_2_ + Ral, the response to E_2_ but not to Ral was up-regulated by about 50%, but when injected together there was no more inhibition of E_2_ by Ral (Figure 10(a)). When Ovx female rats were pretreated similarly but for 1 week instead of 3 days, similar results were obtained, but at higher extent (Figure 10(b)).

## 4. Discussion

The key finding in the present study is that rat female pituitary is a hormone-responsive organ which responds at different stages of development, to**** different estrogenic compounds similar to other rat organs such as the skeleton, the uterus, and the vascular ones [29]. First, E_2_ as well as different phytoestrogens and their carboxy-derivatives stimulate CK activity in rat pituitary of both immature and Ovx similar to other estrogen-responsive organs such as the skeleton and the vasculature ones, both by single and long-term multiple injections [29]. Second, E_2_ as well as different phytoestrogens and their carboxy-derivatives stimulate CK activity in rat pituitary of both immature and Ovx similar to other estrogen-responsive organs such as the skeleton and the vasculature. This response is inhibited by the SERM Ral [29–31]. Third, all phytoestrogens-induced CK were not significantly affected by addition of E_2_, except that of cG and cBA behaved like SERMs and inhibited E_2_ stimulated CK similar to other estrogen-responsive organs such as the skeleton and the vasculature [32]. Fourth, the less-calcemic analogs of vitamin D, JKF and QW, per se increased CK in rat pituitary; moreover, pretreatment with JKF or QW for different time periods at both immature and Ovx rats up-regulated the response and the sensitivity to E_2_ similar to other estrogen-responsive organs such as the skeleton and the vasculature [25, 30]. Fifth, licorice and the phytoestrogens derived from it, that is, glabridin and glabrene also stimulated rat pituitary CK activity from both immature and Ovx rats, when applied for different time periods either by feeding or injections. This stimulation was dose dependent. It is of interest to note that unlike Gla, Glb was also SERM-like and inhibited E_2_ stimulated CK similar to other estrogen-responsive organs such as the skeleton and the vasculature [31]. Sixth, E_2_ failed to stimulate CK activity in pituitary from diabetic immature and Ovx rats, even at increased doses, similar to other estrogen-responsive organs such as the skeleton and the vasculature [23].

 Previous studies showed that the stimulation of CK in the pituitary is similar to other rat organs, such as epiphysis and diapyhsis in the skeleton as well as aorta and left ventricule in the vasculature, but not in the uterus [30].

 This is in accordance with other studies clearly demonstrating that the pituitary is influenced by E_2_ in both immature and Ovx rats, when other biological responses such as hormonal secretion were determined [3]. This is similar to other studies demonstrating the presence of ERs in the rat pituitary, which are responding to E_2_ as well as to phytoestrogens like revestranol [3]. Moreover phytoestrogens were found to influence also brain development, neural function, and behavior parametrs [13].

 The licorice derived phytoestrogens were previously shown by us to have similar effects on the skeleton and the vasculature both in vivo and in vitro in cell derived systems [11, 12, 27, 31].

 The dietary estrogens exert biological activity and affect gonadotropin release from the pituitary [10] via both ER*α* and ER*β* present in the rat pituitary [33], but the present study is showing directly modulation of CK, which is an estrogenic marker, in rat pituitary.

 Vitamin D active metabolites have important physiological effects which are mediated via intracellular receptors which are present in a variety of organs and tissues including rat and human pituitary [34, 35]. Vitamin D also affects hormone secretions in the rat pituitary [36]. Moreover, E_2_ regulates vitamin D-mediated calcium absorption by the induction of cytosolic calcium binding protein (CaBP-D9K) [37] via ERs in the rat pituitary [38]. 

 As previously shown in other organs [30], vitamin D analogs up-regulate the response and the sensitivity of different rat organs from both immature and Ovx rats to E_2_ and to different phytoestrogens. In the present study, the pituitary was shown to have similar properties, suggesting that there is mutual modulation of this hormonal responsiveness in this organ as well.

 We have noticed that in diabetic rats the response to E_2_ of different rat organs from both immature and Ovx rats was abolished compared to intact rats [23]. In the present study, the pituitary was shown to have similar properties, namely, complete abolition of estrogenic response of rat pituitary in the diabetic condition at both types of rats, even when increased hormone levels were injected.

 In conclusion, rat female pituitary is a hormone-responsive organ which responds differently at different stages of development, to a variety of estrogenic compounds similar to other rat organs such as the skeleton and the vasculature but not the uterus. It is important to notice that all compounds were effective in using them at their optimal dose. But in immature female rats E_2_ and G were most effective, and this small difference was not apparent in Ovx female rats. Moreover it also responds to vitamin D analogs alone and to their up-regulation properties. These might suggest taking into consideration the response of the pituitary in addition to the other organs, when hormonal treatment, such as hormone replacement therapy for postmenopausal women, is used for both its beneficial and hazardous aspects.
